# Changes in body mass, appetite-related hormones, and appetite sensation in women during 4 days of hypobaric hypoxic exposure equivalent to 3,500-m altitude

**DOI:** 10.1152/japplphysiol.00369.2022

**Published:** 2022-12-08

**Authors:** Hannes Gatterer, Johanna Roche, Rachel Turner, Giovanni Vinetti, Giulia Roveri, Maja Schlittler, Michael Kob, Anna Walzl, Tomas Dal Cappello, Tadej Debevec, Christoph Siebenmann

**Affiliations:** ^1^Institute of Mountain Emergency Medicine, Eurac Research, Bolzano, Italy; ^2^Institute for Sports Medicine, Alpine Medicine and Health Tourism (ISAG), UMIT TIROL–Private University for Health Sciences and Health Technology, Hall in Tirol, Austria; ^3^Department of Pathophysiology and Transplantation, University of Milan, Milan, Italy; ^4^Division of Clinical Nutrition, Bolzano Regional Hospital, Bolzano, Italy; ^5^Department of Anaesthesiology, University Hospital, LMU Munich, Munich, Germany; ^6^Faculty of Sport, University of Ljubljana, Ljubljana, Slovenia; ^7^Department of Automation, Biocybernetics, and Robotics, Jožef Stefan Institute, Ljubljana, Slovenia

**Keywords:** appetite, food intake, hypoxia, weight management

## Abstract

Altitude exposure may suppress appetite and hence provide a viable weight-loss strategy. While changes in food intake and availability as well as physical activity may contribute to altered appetite at altitude, herein we aimed to investigate the isolated effects of hypobaric hypoxia on appetite regulation and sensation. Twelve healthy women (age: 24.0 ± 4.2 years, body mass: 60.6 ± 7.0 kg) completed two 4-day sojourns in a hypobaric chamber, one in normoxia [P_B_ = 761 mmHg, 262 m (NX)] and one in hypobaric hypoxia [P_B_ = 493 mmHg (HH)] equivalent to 3,500-m altitude. Energy intake was standardized 4 days prior and throughout both sojourns. Plasma concentrations of leptin, acylated ghrelin, cholecystokinin (CCK), and cytokine growth differentiation factor 15 (GDF15) were determined every morning. Before and after breakfast, lunch, and dinner, appetite was assessed using visual analog scales. Body mass was significantly decreased following HH but not NX (−0.71 ± 0.32 kg vs. –0.05 ± 0.54 kg, condition: *P* < 0.001). Compared to NX, acylated ghrelin decreased throughout the HH sojourn (condition × time: *P* = 0.020), while leptin was higher throughout the entire HH sojourn (condition: *P* < 0.001). No differences were observed in CCK and GDF15 between the sojourns. Feelings of satiety and fullness were higher (condition: *P* < 0.001 and *P* = 0.013, respectively), whereas prospective food consumption was lower in HH than in NX (condition: *P* < 0.001). Our findings suggest that hypoxia exerts an anorexigenic effect on appetite-regulating hormones, suppresses subjective appetite sensation, and can induce weight loss in young healthy women. Among the investigated hormones, acylated ghrelin and leptin most likely explain the observed HH-induced appetite suppression.

**NEW & NOTEWORTHY** This study investigated the effects of hypoxia on appetite regulation in women while strictly controlling for diet, physical activity, menstrual cycle, and environmental conditions. In young women, 4 days of altitude exposure (3,500 m) decreases body weight and circulating acylated ghrelin levels while preserving leptin concentrations. In line with the hormonal changes, altitude exposure induces alterations in appetite sensation, consisting of a decreased feeling of hunger and prospective food intake and an increased feeling of fullness and satiety.

## Graphical abstract

**Figure:**
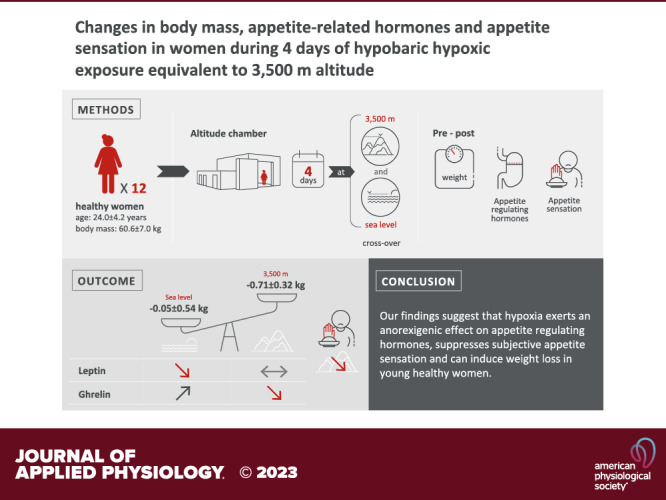

## INTRODUCTION

The global prevalence of overweight and obesity is increasing, warranting the identification and evaluation of new preventive measures or therapies (1). High-altitude exposure has been proposed as a weight-loss strategy (2–5), based on the observations that hypoxia increases basal metabolic rate (BMR) and reduces appetite and thus energy intake (2, 6, 7). While a higher BMR in hypoxia could result from increased sympathetic activity, altered thyroid activity, and increased interleukin 6 (IL-6) levels (2, 7, 8), reduced appetite has been attributed to modification of hormonal appetite regulation (6).

Of the various hormones that could mediate a hypoxia-induced reduction in appetite (6), the orexigenic hormone ghrelin and the anorexigenic agents leptin and cholecystokinin (CCK) have been studied the most (6). Circulating ghrelin indeed seems to be blunted by short-term (<24 h) hypoxic exposure (9, 10), although the more long-term effects are still unclear (6). The effect of hypoxia on leptin is controversial, since leptin has been found to be increased, unaltered, or even decreased at altitude (11–15). Finally, although reductions in CCK have been reported at altitude (10, 16), these reductions were modest and not a universal finding (6). In addition to these hormones, the stress response cytokine growth differentiation factor 15 (GDF15) has recently gained attention due to its potent suppressive effects on food intake and body weight (17, 18). GDF15 has been shown to increase at altitude (19), but whether this increase affects appetite sensation and food intake at altitude has not been studied.

Taken together, the effects of hypoxia on appetite and the underlying regulation are not fully elucidated and to some extent remain controversial (6). It needs to be considered that appetite-regulating hormones may be influenced at altitude not only by hypoxia but also by changes in physical activity, food, and fluid intake (6, 7, 20). Moreover, biological sex and female sex hormones may influence appetite regulation and energy metabolism (21). Since former studies either did not include women or did not account for potential variations induced by the menstrual cycle, studying the effect of altitude on appetite in women while controlling for the menstrual cycle seems particularly warranted. The aim of this study was therefore to investigate the isolated effects of hypoxia on appetite-regulating hormones and appetite sensation in women by tightly controlling for these confounding factors.

## METHODS

### Participants

Twelve healthy women (age: 24.0 ± 4.2 yr; body mass: 60.6 ± 7.0 kg; height: 167.5 ± 7.5 cm; and body mass index: 21.5 ± 2.4 kg/m^2^) gave written informed consent to participate in the crossover study. All participants lived close to sea level and had no history of high altitude-related illnesses. They were instructed to refrain from visiting altitudes >2,000 m in the month preceding and throughout the study. Participants did not perform regular endurance exercise but were recreationally active one to two times a week (e.g., climbing, walking, mountain biking). To minimize variations induced by the menstrual cycle, only participants using a monophasic hormonal contraception were recruited and study visits were scheduled during the identical time points of the contraception cycle (i.e., during the active phase of pill consumption). The study was approved by the ethics committee of the Bolzano Hospital, Italy (No. 70-2019) and conducted in agreement with the Declaration of Helsinki (except registration in a clinical database, as we consider this a physiological and not a clinical study).

### Study Protocol

The experiments were conducted as part of a project investigating different physiological effects of 4 days of hypoxia in women (22). Participants completed two 4-day sojourns in a hypobaric chamber (terraXcube, Bolzano, Italy), with barometric pressure either unmodified [normoxia (NX), P_B_ = 761 mmHg, 262 m] or reduced to the equivalent of ∼3,500-m altitude [hypobaric hypoxia (HH), P_B_ = 493 mmHg). The study followed a crossover design and the sojourns were separated by a 4-week washout period. Participants were not blinded to the condition and were allocated to the sojourn order according to their availability and contraception calendar. Temperature and humidity were identical between the two sojourns (22°C and 30%, respectively). Both sojourns started in the early morning with decompression corresponding to an ascent rate of 2 m/s (for the HH sojourn). Food and water intake were standardized during a 4-day lead-in period preceding the sojourns and throughout the sojourns. Physical activity was kept the same during both sojourns (details below). Throughout both stays, participants studied or enrolled in recreational activities and spent the time between 11 PM and 7 AM in bed.

### Procedures and Measurements

#### Standardized diet, physical activity, and body weight measurement.

The standardized diet consumed during the 4-day lead-in period and throughout the sojourns was identical between NX and HH. Daily food intake consisted of three main meals and three snacks. While main meals changed daily, they were always the same between NX and HH for a given lead-in or sojourn day. The daily caloric intake was set at ∼1,900 kcal (72 g (18%) proteins, 239 g (61%) carbohydrates, and 81 g (21%) fat). The 1,900 kcal/day were derived from the calculation of the basal metabolic rate for women aged 18–29 yr according to the Schofield equation (23) and using a physical activity level factor of 1.45, which corresponds to a sedentary lifestyle (24). The use of this equation is recommended by the Italian Society for Human Nutrition (SINU) (25). Sodium and potassium intake were ∼2,200 and 2,300 mg/day, respectively, and iron intake ∼9 mg/day. Participants drank 2 liters of water per day distributed into six equal portions that were consumed with the meals/snacks. All participants consumed all the food and fluid provided during the two lead-in periods and the two-chamber sojourns. During the first lead-in period and sojourn, participants were allowed to increase the size of the snacks if they wished, but none of the participants took advantage of this offer.

During the 4-day lead-in period preceding the first sojourn, participants were asked to follow their normal daily routines and physical activity was recorded with a step counter. During the sojourns, participants replicated the average number of daily steps performed during the lead-in on a treadmill (i.e., 6,324 ± 2,264 steps/day during the NX and 6,245 ± 2,242 steps/day during the HH sojourn, *P* = 0.07) and were allowed to perform light strength training and stretching. Running on the treadmill was not allowed. Participants were free to complete the steps whenever they wanted during the day but were advised not to perform all steps in a single session and to distribute them over the day.

Body mass was measured to the nearest 0.05 kg (PS 240, Beurer, Ulm, Germany) daily after the first voiding and before breakfast with the participants always wearing the same clothes.

#### Blood sampling and analyses.

In the morning before the start of the sojourns (6 AM, referred to as “Pre”), and then on each morning of the sojourns (7 AM), blood was drawn from the antecubital vein while participants were fasted and still in bed. After blood collection, the monovette tubes were immediately placed on ice and centrifuged for 15 min at 1,600 *g* and 4°C. The plasma concentrations of acylated ghrelin and CCK were measured on all these time points, whereas leptin and GDF15 concentrations were only determined Pre and after the first and fourth night (morning 1 and 4, respectively).

Leptin and acylated ghrelin concentrations were measured in plasma derived from EDTA tubes using ELISA [leptin ELISA (IBL International GMBH, Germany), sandwich assay using two specific and high-affinity antibodies] and enzyme immunometric assay [acylated ghrelin (human; Bertin Pharma, Montigny-le-Bretonneux, France, double-antibody sandwich technique], respectively. For the determination of acylated ghrelin, a protease inhibitor (AEBSF protease inhibitor, ThermoFisher, Bologna, Italy) was added to the EDTA blood. GDF15 levels were determined using the Human GDF15 ELISA (Novatein Biosciences, solid-phase sandwich enzyme immunoassay technique) and CCK levels using human CCK ELISA kit ELISA (Novatein Biosciences, biotinylated CCK spiked into the samples or standards).

Fasting glucose and lactate concentration were determined each morning from the blood gas analysis (ABL90 FLEX, Radiometer, Copenhagen, Denmark).

#### Appetite sensation.

Before and after breakfast, lunch, and dinner, appetite was assessed by visual analog scales (VAS) as specified elsewhere (26). Briefly, the VAS was used to record hunger, satiety, fullness, prospective food consumption, and the desire to eat something sweet, salty, savory, or fatty. The scales were composed of a 100-mm line with words anchored at each end and describing the extremes (i.e., “I am not hungry at all” – ” I have never been more hungry” for the question “how hungry do you feel?”; “I am completely empty” – “I cannot eat another bite”, for the question “how satisfied do you feel?”; “not at all full” – “totally full”, for the question “how full do you feel?”; “nothing at all” – “a lot”, for the question “how much do you think you can eat?” and for the questions whether you would like to eat something sweet, salty, savory or fatty “yes, very much – no, not at all”) and participants were instructed to make a mark across the line corresponding to their sensation (26). Quantification of the measurement was done by measuring the distance from the left end of the line to the mark.

#### Acute mountain sickness.

Acute mountain sickness (AMS) symptoms were evaluated with the Lake Louise AMS Scoring system (LLS) the first evening and thereafter every morning of the sojourns. The LLS evaluates the severity of a headache, gastrointestinal symptoms, fatigue/weakness, dizziness/light-headedness, and sleep, rated on a scale of severity from 0 to 3 (27).

### Statistical Analyses

Generalized estimating equations (GEE) were performed to analyze appetite-regulating hormones (acylated ghrelin, CCK, GDF15, and leptin), body weight, variables assessing appetite sensation, LLS, sleep quality, glucose and lactate levels, and sodium concentration. The factors investigated were condition (NX vs. HH), time (days of sojourns), the order of the sojourns (first HH and then NX or vice versa), and the interaction between condition and time. For the variables assessing appetite sensation, the time point during the day (before and after breakfast, lunch, and dinner) was also analyzed, while for the appetite-regulating hormones, glucose, lactate, and sodium concentration and the body weight the respective Pre values were inserted in the GEE as covariates. CCK, GDF15, body weight, glucose, lactate, and sodium levels, and variables assessing appetite sensation were normally distributed. Acylated ghrelin and leptin followed a gamma distribution, while for the LLS and the sleep quality, the Poisson distribution was used. In the GEE, the specified link function of the normally distributed parameters was identity, while logarithmic transformation was used for the Gamma and Poisson distribution. Normal distribution of the data was assessed by means of Shapiro-Wilk test and normal Q-Q plots. All available data were included in the statistical analysis (i.e., *n* = 11 in NX and *n* = 12 in HH) and GEE estimated the missing data. To compare Pre values between the NX and the HH sojourns, paired *t* test and Wilcoxon signed-rank test were used, as appropriate. *P* values were adjusted for a false discovery rate of 0.05 using the Benjamini-Hochberg method. SPSS version 27 (IBM Corp., Armonk, NY) was used for statistical analysis, and *P* < 0.05 (two-sided) was considered statistically significant. Values are reported as means ± SD.

## RESULTS

**Figure 1.**
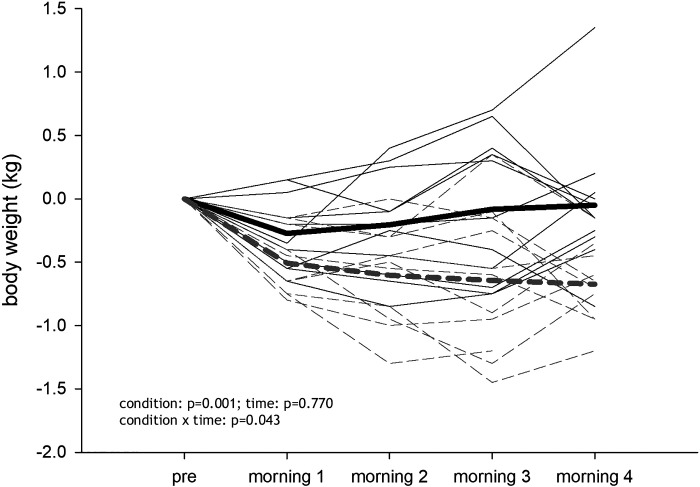
Body mass changes over the course of the hypobaric hypoxia (HH) and normoxia (NX) sojourn (differences from Pre are shown). Gray dashed line shows the HH sojourn; *n* = 11.

One participant did not participate in the NX sojourn (private reasons), and one participant left the chamber on the last evening of the HH sojourn for medical reasons that were, to the best of our knowledge, unrelated to the study interventions. The mean LLS was 0.53 ± 0.82 during the HH sojourn and 0.25 ± 0.32 during the NX sojourn and decreased with time (condition: *P* = 0.217; time: *P* < 0.001; condition × time: *P* = 1.000, values are presented in Table 1). The nausea score was 0 for all participants at every time point, except for one participant reporting 1 (mild nausea) on the first evening in HH. Sleep quality according to the LLS did not differ between sojourns and improved with time (condition: *P* = 0.061; time: *P* < 0.001; condition × time: 0.932, Table 1). Fasting glucose levels did not change in NX but decreased in HH (condition: *P* = 0.259; time: *P* < 0.001; condition × time: *P* < 0.001; effect of Pre levels: *P* < 0.001), whereas fasting lactate levels tended to be higher in HH compared to NX (condition: *P* = 0.051; time: *P* = 0.195; condition × time: *P* < 0.001; effect of Pre levels: *P* = 0.255, Table 1). During the HH sojourn body weight decreased by -0.71 ± 0.32 kg, whereas a reduction of -0.05 ± 0.54 kg was observed in NX (condition: *P* = 0.001; time: *P* = 0.770; condition × time: *P* = 0.043; effect of Pre body weight: *P* < 0.001; Fig. 1). An effect of the order of the sojourns was detected for LLS (*P* = 0.029), sleep quality (*P* = 0.049), and glucose levels (*P* = 0.044).

**Table 1.** Sodium, glucose, and lactate concentration as well as sleep quality and the Lake Louise Score in the course of the hypobaric hypoxia and the normoxia sojourn

Parameter/Condition	Time					*P* Value*	
	Pre	Morning 1	Morning 2	Morning 3	Morning 4	Condition	Condition × time
Sodium, mmol/L,							
NX	141.5 ± 1.2	140.7 ± 1.4	141.2 ± 1.3	141.0 ± 1.3	141.4 ± 1.8	0.715	0.003
HH	140.6 ± 1.2	140.6 ± 1.4	140.2 ± 1.1	140.7 ± 1.9	140.8 ± 1.7		
Glucose, mg/dL							
NX	86.6 ± 3.5	87.3 ± 2.8	88.0 ± 3.0	86.9 ± 3.1	86.4 ± 2.5	0.259	<0.001
HH	87.3 ± 4.9	88.6 ± 5.9	86.1 ± 4.0	84.0 ± 4.3	85.3 ± 5.1		
Lactate, mmol/L							
NX	1.0 ± 0.5	0.8 ± 0.4	1.0 ± 0.4	0.9 ± 0.5	1.1 ± 0.8	0.051	<0.001
HH	0.9 ± 0.4	1.2 ± 0.5	0.9 ± 0.4	1.4 ± 0.7	1.1 ± 0.5		
Lake Louise Score							
NX		0.6 ± 1.0	0.1 ± 0.3	0.2 ± 0.4	0.1 ± 0.3	0.217	1.000
HH		1.0 ± 1.0	0.3 ± 0.7	0.4 ± 0.9	0.2 ± 0.6		
Sleep quality							
NX		0.4 ± 0.5	0.0 ± 0.0	0.1 ± 0.3	0.1 ± 0.3	0.061	0.932
HH		0.8 ± 0.8	0.3 ± 0.7	0.4 ± 0.9	0.2 ± 0.6		

Sleep quality was assessed according to the Lake Louise Score. HH, hypobaric hypoxia; NX, normoxia. *Calculated with generalized estimating equations and adjusted by means of Benjamini-Hochberg method.

### Appetite-Related Hormones

**Figure 2.**
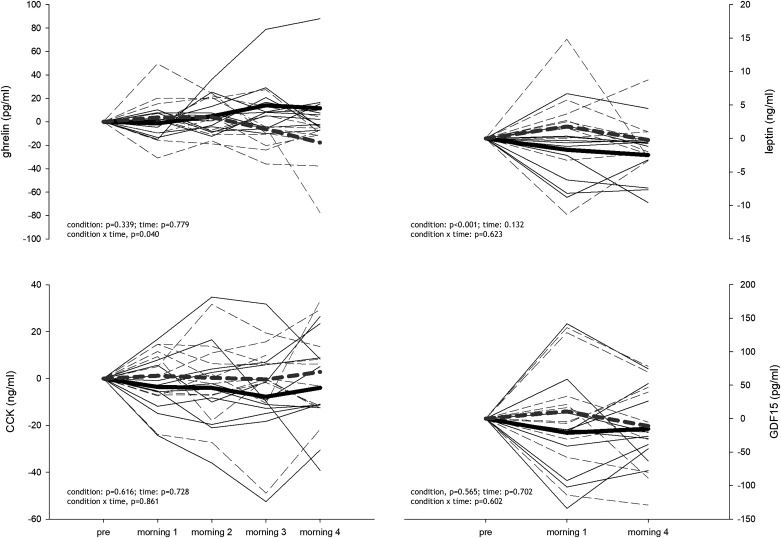
Fasting acylated ghrelin, leptin, growth differentiation factor 15 (GDF15) and cholecystokinin (CCK) changes over the course of the hypobaric hypoxia (HH) and normoxia (NX) sojourn (differences from Pre are shown). Gray dashed line shows the HH sojourn; *n* = 11.

Pre values of appetite-regulating hormones were similar between sojourns (Table 2). Changes in hormones from the Pre values are presented in Fig. 2. Compared to NX, acylated ghrelin decreased throughout the HH sojourn (condition: *P* = 0.339; time: *P* = 0.779; condition × time, *P* = 0.040, Fig. 2, *top left*), while leptin levels were higher throughout the entire HH sojourn (condition: *P* < 0.001; time: *P* = 0.132; condition × time: *P* = 0.623, Fig. 2, *top right*). Conversely, CCK (condition: *P* = 0.616; time: *P* = 0.728; condition × time: *P* = 0.861, Fig. 2, *bottom left*) and GDF15 (condition: *P* = 0.565; time: *P* = 0.702; condition × time: *P* = 0.602, Fig. 2, *bottom right*) were not different between the sojourns. There was an effect of the Pre value for all appetite-regulating hormones (*P* = 0.009 for CCK and *P* < 0.001 for all the others) and an effect of the order of the sojourns for CCK (*P* = 0.044).

**Table 2.** Body mass and fasting appetite-regulating hormones before the hypobaric hypoxia and the normoxia sojourn

	Pre HH	Pre NX	*P* Values Adjusted
Body mass, kg	60.6 ± 7.0	60.6 ± 7.3	0.734
Ghrelin, pg/mL	57.1 ± 25.6	37.0 ± 28.5	0.131
Leptin, ng/mL	18.9 ± 15.9	18.5 ± 16.6	1.000
GDF15, pg/mL	323.5 ± 124.2	301.6 ± 121.4	0.798
CCK, ng/mL	26.3 ± 16.3	31.1 ± 19.4	0.694

For body weight, growth differentiation factor 15 (GDF15) and cholecystokinin (CCK) paired *t* tests were used, while for acylated ghrelin and leptin Wilcoxon signed-rank tests were run. *P* values were adjusted by means of the Benjamini-Hochberg method. HH, hypobaric hypoxia; NX, normoxia.

### Appetite Sensation and Food Preference

**Figure 3.**
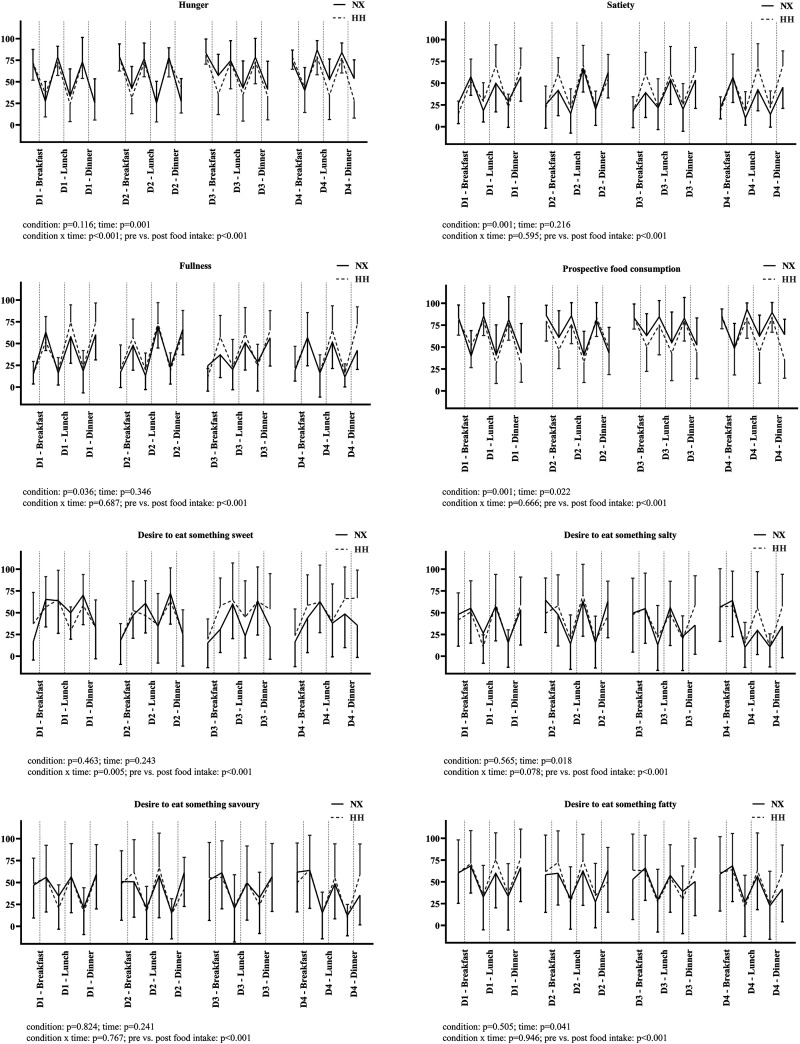
Appetite sensation before and after the meals during both sojourns. Data are presented as mean ± SD; *n* = 11 for normoxia (NX) and *n* = 12 for hypobaric hypoxia (HH).

Figure 3 illustrates the perception of hunger and satiety as well as food preferences. Feelings of hunger increased during NX, but not during the HH sojourn (condition: *P* = 0.116; time: *P* = 0.001; condition × time: *P* < 0.001; Pre vs. Post food intake: *P* < 0.001, Fig. 3, *row 1*, *left*). Prospective food consumption was lower in HH and increased during the sojourns (condition: *P* = 0.001; time: *P* = 0.022; condition × time: *P* = 0.666; Pre vs. Post food intake: *P* < 0.001, Fig. 3, *row 2*, *right*). Similarly, the feeling of satiety and fullness were higher in HH (condition: *P* = 0.001 and *P* = 0.036; time: *P* = 0.216 and *P* = 0.346; condition × time: *P* = 0.595 and *P* = 0.687; Pre vs. Post food intake: *P* < 0.001 and *P* < 0.001, respectively, Fig. 3, *row 1*, *right* and *row 2*, *left*). The desire to eat something sweet was decreased in NX, whereas it tended to be increased in HH (condition: *P* = 0.463; time: *P* = 0.243; condition × time: *P* = 0.005; Pre vs. Post food intake: *P* < 0.001, Fig. 3, *row 3*, *left*). The desire to eat something salty, savory, and fatty was not different between the sojourns (condition: *P* = 0.565, *P* = 0.824 and *P* = 0.505; time: *P* = 0.018, *P* = 0.241 and *P* = 0.041; condition × time: *P* = 0.078, *P* = 0.767 and *P* = 0.946; Pre vs. Post food intake: *P* < 0.001, *P* < 0.001 and *P* < 0.001, respectively, Fig. 3, *row 3*, *right* and *row 4*). An effect of the order of the sojourns was detected for the feelings of hunger (*P* < 0.001), satiety (*P* = 0.007), and fullness (*P* = 0.001), for prospective food consumption (*P* < 0.001), and for the desire to eat something salty (*P* = 0.005) and savory (*P* = 0.020).

## DISCUSSION

To our knowledge, this is the first study investigating the effects of hypoxia on appetite regulation in women while strictly controlling for diet, physical activity, menstrual cycle, and environmental conditions. Comparison of the effects of two identical sojourns, one in NX and one in HH, confirms that exposure to HH equivalent to an altitude of 3,500 m reduces circulating acylated ghrelin and preserves circulating leptin concentrations, respectively. In line with these hormonal changes, we observed that HH exposure induces alterations in appetite sensation, consisting of a decreased feeling of hunger and prospective food intake, and an increased feeling of fullness and satiety.

Altitude exposure has been proposed as a potential weight-loss strategy (2–5). However, the effects of hypoxia on appetite and weight loss remain controversial, potentially as they depend on the severity of hypoxia, the duration of exposure, the level of physical activity, and access to food (20). Regarding the severity, the effects of moderate altitude (1,500–3,500 m) in particular are unclear, even though these altitudes presumably have the most therapeutic potential (7) as they are easy to reach and carry little risk of facilitating altitude sickness and other hypoxia-related complications. The present results support that, at least in females, such moderate altitudes are sufficient to exert significant effects on appetite-regulating hormones and appetite sensation and may induce body weight loss. HH reduced acylated ghrelin and preserved leptin levels and, presumably as a consequence, attenuated the sensation of appetite. Acylated ghrelin exerts orexigenic effects and was reported to be a particularly sensitive marker of altered appetite signaling at altitude (6, 9, 28). While the mechanisms for suppression of acylated ghrelin at altitude remain unclear, the inhibition of the posttranslational acylation of ghrelin, decreased availability of medium-chain fatty acids as the substrate for acylation, or inhibited secretion from P/D1 cells may be involved (28). It needs to be noted that acylated ghrelin levels were somewhat lower at Pre HH than at Pre NX (Table 1). Although this difference was not statistically significant, these data should therefore be considered with caution (for instance the morning 4 values did not differ between HH and NX: 42.7 ± 19.2 vs. 52.3 ± 35.6 pg/mL, respectively, *P* = 0.488; paired sample *t* test). With regard to leptin, body weight loss and low food availability have been shown to reduce leptin levels (29). Our data indicate that HH may counteract this process, potentially reducing the feeling of hunger at altitude. The mechanisms for the increase in leptin levels at altitude are not fully elucidated, but HIF pathways seem to be involved (6). It should be noted that sleep is often disturbed at altitude, which cannot only affect ghrelin but also other hormones involved in appetite sensation (e.g., leptin) (30). However, this is unlikely to explain our findings since sleep quality assessed by the LLS did not differ between sojourns.

In contrast to acylated ghrelin and leptin, GDF15, which due to its potent suppressive effects on food intake and body weight could have been a promising candidate involved in appetite regulation at altitude (17, 19), seems unaffected by HH, at least by the applied severity or in the population studied. Similarly CCK, does not seem to play an important role in HH-related appetite modulation, which is in line with previous evidence (6). Further studies, however, need to determine whether more severe or prolonged altitude exposures can modulate these hormones.

Intriguingly, in HH the desire to eat something sweet increased throughout the sojourn, whereas in NX both the desires to eat something sweet and something salty decreased. These observations are in line with a study showing that acute altitude exposure increases the preference for sweet food (31). The mechanisms behind these changes are still unclear but may be linked to an acute metabolic switch towards glucose metabolism at altitude (32). Of note, we detected small differences in fasting glucose (i.e., decreased at altitude) and lactate (i.e., higher at altitude) between the HH and the NX sojourn (Table 2). Such a switch could support acclimatization to the hypoxia at altitude, since enhanced glucose dependency increases the energy yield per unit oxygen consumed (33). However, given the small differences, a direct influence on appetite seems unlikely.

An unexpected finding was a reduction in body weight after only 4 days of HH. This may have been partly attributed to an increased BMR, since the same amount of food and fluid was provided. While we did not determine BMR, others have reported BMR to increase by 10–28% during the first days at altitudes between 3,500–5,000 m (2, 34). Assuming a normal BMR of ∼1,400 kcal/day (FAO/WHO/UNU equation; Ref. 35), this would have increased the daily calorie expenditure by 140–400 kcal. Assuming a resting respiratory exchange ratio of 0.85 (36) reflecting a mixed substrate (50% fat, 50% carbohydrates/proteins) utilization, this would have induced an estimated fat loss of 8–22 g/day (1 g fat = 9 kcal; Ref. 37). Additionally, some of the body weight loss may have reflected the loss of fat-free mass (FFM) including body water, although the latter must have been minimal, given that as reported elsewhere (22), 24-h urine volumes were almost identical between sojourns (2,135 ± 142 vs. 2,130 ± 153 mL/day, for NX and HH, respectively *P* = 0.915), and changes in total body water throughout the sojourns were minimal (−0.108 liters vs. 0.003 liters in HH and NX, respectively; *P* = 0.347 *n* = 8) in our participants. Finally, intestinal malabsorption could have contributed to a negative energy balance in HH, although this seems unlikely as malabsorption is only described at much higher altitudes (7). It is noteworthy that men previously exposed to the same study protocol experienced no HH-induced reduction in body weight (38, 39), suggesting a possible sex-specific effect of HH on body weight that could be investigated in future studies.

### Methodological Considerations

Even though Pre acylated ghrelin levels were not statistically different when adjusted for a false discovery rate of 0.05, the numerical difference needs further consideration (unadjusted *P* = 0.026). Given that the lead-in periods before the sojourns were standardized and various potential confounders were controlled for (e.g., food intake, menstrual cycle), the differences are difficult to explain and suggest that factors not considered in our protocol can influence acylated ghrelin. Altered physical activity levels might be one factor, yet this seems unlikely as acylated ghrelin levels were reported unaffected even the day after performing high-intensity exercise (40). Moreover, participants were advised not to change their physical activity habits during the entire study period. Additionally, insufficient sleep, as mentioned earlier, could be another factor, as sleep deprivation for only 2 days was reported to increase ghrelin (41). Thus, it could be speculated that especially before the first sojourn, feelings related to study anticipation might have influenced sleep quality and thus acylated ghrelin. However, since Pre values of acylated ghrelin and leptin of the first and second study blocks were not different (48.6 ± 30.1 vs. 45.5 ± 27.9, *P* = 0.534 and 18.0 ± 16.2 vs. 19.4 ± 16.3, *P* = 0.424 for acylated ghrelin and leptin, respectively), such an impact of sleep quality seems unlikely. Nonetheless, the observation that despite controlling for multiple confounding factors, baseline differences in acylated ghrelin can occur needs attention and consideration in future studies. Moreover, it needs to be noted that hormones were not assessed in the fed state; thus we were not able to identify a potential hypoxia effect on postprandial appetite-related hormonal modulation. It also needs to be acknowledged that the study was not designed to determine body composition; therefore, it is not possible to establish whether and how much of the body weight loss is due to fat mass or FFM loss. Moreover, due to the relatively short observation period (i.e., 96 h), long-term hypoxic effects on metabolic homeostasis cannot be addressed.

### Practical Relevance and Conclusions

The present data, collected under strictly controlled conditions, demonstrate that short-term HH exposure (i.e., 4 days at 3,500 m) exerts an effect on appetite-regulating hormones (i.e., anorexigenic effect), suppresses appetite sensation, and can induce body weight loss in young healthy females. The magnitude of the observed weight loss was somewhat unexpected, as the participants received a standardized diet during both sojourns and no such effect was found in males (38, 39). Among the investigated hormones, acylated ghrelin and leptin were the most likely to explain the effect on appetite suppression. These findings are promising when considering hypoxic exposure as a therapeutic agent for weight management. However, it remains to be determined whether the modest changes observed in these hormones are sufficient to affect ad libitum food intake. Furthermore, it should be noted that especially in individuals with overweight and obesity, the ability of leptin to inhibit appetite can be blunted (42). Therefore, transferring the present findings to those with obesity needs to be done with caution. Nonetheless, it has been reported that even in individuals with obesity, 1 wk of moderate altitude exposure increases leptin levels and induces weight loss (43) supporting transferability. From a practical perspective, it should furthermore be considered whether the observed beneficial effects could also be induced by shorter hypoxic exposures (e.g., intermittent hypoxic gas breathing). Such short-term normobaric intermittent hypoxia exposure (e.g., $\text{F}{\text{I}}_{{\text{O}}_{2}}$ 12–14%, 2 times a week for 180 min), however, seems not to confer comparable benefits (5). In sum, the present results support the potential of HH exposure to induce weight loss in young healthy females. Further studies should establish whether women with obesity show a similar hypophagic response to moderate altitude exposure and whether this response is maintained or otherwise with acclimatization. Equally, whether there are indeed sex differences in hormonal regulation and adaptation of appetite sensation with isolated HH exposure remains to be determined.

## DATA AVAILABILITY

Data will be made available upon reasonable request.

## GRANTS

This work was funded by The Swiss National Center of Competence in Research (NCCR) Kidney Control of Homeostasis (Kidney.CH) and the Herbert N. Hultgren Grant (Wilderness Medical Society, USA).

## DISCLOSURES

No conflicts of interest, financial or otherwise, are declared by the authors.

## AUTHOR CONTRIBUTIONS

H.G., M.K., T.D., and C.S., conceived and designed research; H.G., J.R., R.T., G.V., G.R., A.W., and C.S. performed experiments; H.G. and T.D.C. analyzed data; H.G. and C.S. interpreted results of experiments; H.G., T.D.C., and M.S. prepared figures; H.G. and C.S. drafted manuscript; H.G., J.R., R.T., G.V., G.R., M.S., M.K., A.W., T.D.C., T.D. and C.S. edited and revised manuscript; H.G., J.R., R.T., G.V., G.R., M.S., M.K., A.W., T.D.C., T.D., and C.S. approved final version of manuscript.
